# Kampo medicine as a nutrition-relevant multi-target intervention for sarcopenia: implications for appetite, mitochondrial function, and the frailty-sarcopenia-cachexia continuum

**DOI:** 10.3389/fnut.2026.1871481

**Published:** 2026-08-13

**Authors:** Taneomi Kurokawa, Tomohiro Kurokawa

**Affiliations:** 1 Department of Surgery I, National Defense Medical College, Tokorozawa, Saitama, Japan; 2 Jyoban Hospital of Tokiwa Foundation, Iwaki, Fukushima, Japan; 3 Department of Medical Epigenomics Research, Fukushima Medical University, Fukushima, Japan

**Keywords:** appetite, cachexia, frailty, gut–muscle axis, Kampo medicine, mitochondria, nutritional intake, safety

## Abstract

**Background:**

Sarcopenia is sustained not only by age-related declines in muscle anabolism but also by reduced nutritional intake, anorexia, metabolic dysregulation, mitochondrial dysfunction, chronic inflammation, neuromuscular deterioration, and physical inactivity. Accordingly, interventions that influence appetite, oral intake, nutrient handling, fatigue, metabolic resilience, and physical function may be relevant within multimodal sarcopenia care. Kampo medicine, a traditional Japanese system of standardized multi-component herbal formulations, may be positioned as a nutrition-relevant multi-target intervention; however, its clinical evidence base for sarcopenia remains heterogeneous and largely indirect.

**Methods:**

We conducted a narrative review of published clinical, translational, and mechanistic literature addressing sarcopenia, frailty, cachexia, nutrition-related muscle wasting, the gut–muscle axis, systems pharmacology, and representative pharmaceutical-grade Kampo formulations. Searches were performed in PubMed, Scopus, and Web of Science from database inception to May 2026 using prespecified combinations of disease-, mechanism-, and formula-specific terms, supplemented by an additional search for Juzentaihoto/Juzen-taiho-to and related transliterations in response to peer review. Evidence was interpreted according to whether it directly evaluated sarcopenia outcomes or indirectly addressed nutrition-relevant domains such as appetite, fatigue, inflammation, mitochondrial function, physical activity, or neuromuscular symptoms.

**Results:**

Sarcopenia shares important pathophysiological features with frailty and cachexia, including chronic inflammation, impaired mitochondrial function, anabolic resistance, reduced food intake, fatigue, and neuromuscular dysfunction. Several Kampo formulations, including Ninjin’yoeito, Rikkunshito, Hochuekkito, Juzentaihoto, Hachimijiogan, and Goshajinkigan, have been associated with biological or clinical domains potentially relevant to appetite regulation, oral intake, inflammatory balance, gut microbiota-related pathways, metabolic resilience, and functional support. Nevertheless, direct evidence using standardized sarcopenia criteria and validated endpoints remains limited.

**Conclusion:**

Kampo medicine is best regarded as a hypothesis-generating, nutrition-relevant complementary approach within multimodal sarcopenia care rather than as an established stand-alone therapy for muscle loss. Future studies should use standardized diagnostic criteria, prespecified sarcopenia endpoints, rigorous safety monitoring, and transparent reporting to determine whether selected Kampo formulations can improve clinically meaningful nutrition-related and muscle-related outcomes.

## 1 Introduction

Sarcopenia is a major and growing clinical challenge in aging societies worldwide. The condition is characterized by progressive loss of skeletal muscle strength, muscle quantity or quality, and physical performance, and it contributes to reduced mobility, falls, disability, hospitalization, and mortality among older adults (1–6). In clinical practice, however, skeletal muscle decline is rarely driven by impaired muscle anabolism alone. Reduced oral intake, anorexia, fatigue, metabolic dysregulation, physical inactivity, chronic inflammation, and chronic disease commonly interact with aging biology to accelerate functional decline (2–5, 7–9).

Sarcopenia also overlaps clinically and biologically with frailty and disease-associated cachexia (5, 10, 11). Frailty is characterized by reduced physiological reserve and increased vulnerability to stressors, whereas cachexia is a disease-associated wasting syndrome driven by systemic inflammation, metabolic disturbance, and reduced food intake (7, 8, 10, 11). These conditions are not identical, but they frequently coexist and share important nutrition-related drivers. In older or chronically ill patients, anorexia, inadequate intake, fatigue, inflammation, and poor activity tolerance may determine whether nutritional support and rehabilitation can be maintained.

**Figure 1:**
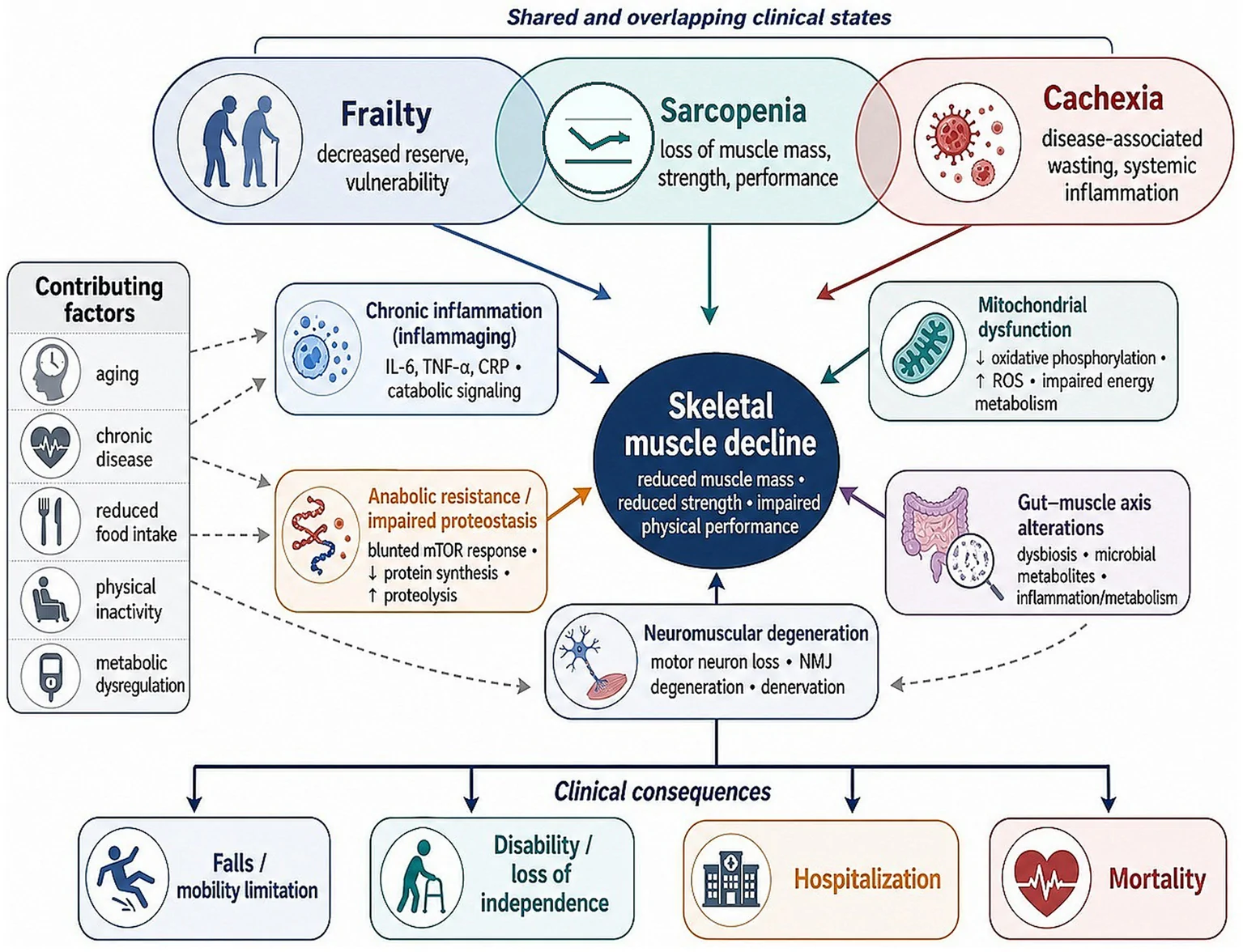
Nutritional and metabolic drivers of skeletal muscle decline across the frailty-sarcopenia-cachexia continuum. Aging, chronic disease, reduced food intake, physical inactivity, metabolic dysregulation, chronic inflammation, mitochondrial dysfunction, anabolic resistance, neuromuscular degeneration, and gut–muscle axis alterations collectively contribute to reductions in muscle mass, strength, and physical performance. The figure emphasizes shared and overlapping clinical states while preserving the conceptual distinction among frailty, sarcopenia, and cachexia. The sarcopenia icon was revised to indicate loss, maintenance, and restoration toward baseline rather than supraphysiological muscle enhancement.

The biology of sarcopenia involves complex interactions among chronic inflammation, mitochondrial dysfunction, anabolic resistance, hormonal change, reduced nutrient intake, and neuromuscular degeneration (3, 12–17) (Figure 1). Because these mechanisms interact within interconnected biological networks, therapeutic strategies directed at a single molecular target may have limited efficacy (18–21). Multimodal interventions that support nutrition, exercise, rehabilitation, and treatment of underlying disease are therefore central to contemporary sarcopenia management (5, 21, 22).

Kampo medicine is a traditional Japanese medical system derived from classical East Asian medicine and adapted into a standardized pharmaceutical framework in modern Japan (23). In the context of sarcopenia, Kampo formulations are of interest not because they have been established as direct anabolic therapies, but because several formulas have been used clinically in patients with anorexia, fatigue, frailty, constitutional weakness, peripheral symptoms, and functional decline. These domains are closely linked to nutritional intake, metabolic resilience, and muscle-related outcomes. Accordingly, Kampo medicine may be better understood as a nutrition-relevant complementary intervention that could support selected domains of multimodal sarcopenia care.

Recent formula-specific reviews in Traditional & Kampo Medicine have summarized the historical background, crude-drug composition, pharmacology, clinical studies, and case literature for frequently used Kampo prescriptions, including Ninjin’yoeito and Rikkunshito (24, 25). These reviews provide an important background framework for international readers. The present review builds on that literature by focusing specifically on nutrition-related muscle-wasting phenotypes, the frailty-sarcopenia-cachexia continuum, evidence proximity to validated sarcopenia endpoints, and translational mechanisms linking appetite, inflammation, mitochondrial function, and the gut–muscle axis.

This review examines Kampo medicine as a nutrition-relevant multi-target intervention for sarcopenia and related muscle-wasting disorders. The central objective is not to claim that Kampo is an established treatment for sarcopenia, but to evaluate the strength and limitations of current evidence, distinguish direct evidence from indirect biological plausibility, and identify priorities for future translational studies.

## 2 Approach to literature review

This article is a narrative review rather than a systematic review or meta-analysis. Nevertheless, in response to the need for methodological transparency in narrative evidence synthesis, we prespecified the literature sources, search concepts, and selection principles used for this review.

We searched PubMed, Scopus, and Web of Science from database inception to May 2026. Search terms were combined across four domains: (i) disease and phenotype terms, including ‘sarcopenia’, ‘frailty’, ‘cachexia’, ‘muscle wasting’, ‘skeletal muscle decline’, ‘older adults’, and ‘anorexia of aging’; (ii) nutrition and mechanism terms, including ‘appetite’, ‘oral intake’, ‘nutrition’, ‘protein intake’, ‘mitochondrial dysfunction’, ‘anabolic resistance’, ‘inflammation’, ‘interleukin-6’, ‘tumor necrosis factor-alpha’, ‘mTOR’, ‘ubiquitin-proteasome system’, ‘gut–muscle axis’, ‘microbiota’, ‘short-chain fatty acids’, and ‘bile acid signaling’; (iii) intervention terms, including ‘Kampo medicine’, ‘traditional Japanese medicine’, ‘herbal medicine’, ‘systems pharmacology’, and ‘network pharmacology’; and (iv) formula-specific terms, including ‘Ninjin’yoeito’, ‘Rikkunshito’, ‘Hochuekkito’, ‘Juzentaihoto’, ‘Juzen-taiho-to’, ‘JTT’, ‘Sipjeondaebo-tang’, ‘Shi-Quan-Da-Bu-Tang’, ‘Hachimijiogan’, and ‘Goshajinkigan’. Reference lists of key reviews and relevant clinical studies were also hand-searched. In response to peer review, a supplementary targeted search was performed for Juzentaihoto and for recent review literature in Traditional & Kampo Medicine on frequently used Kampo prescriptions.

Eligible literature included peer-reviewed clinical studies, observational studies, randomized or non-randomized intervention studies, experimental mechanistic studies, pharmacological studies, and high-quality narrative or systematic reviews relevant to sarcopenia, frailty, cachexia, nutrition-related muscle wasting, Kampo medicine, or the gut–muscle axis. Because the focus of this review is translational relevance to clinical sarcopenia care, particular attention was given to studies reporting appetite, food intake, body composition, muscle strength, physical performance, fatigue, inflammatory markers, quality of life, or adverse events.

Studies were excluded from emphasis if they focused on non-standardized herbal mixtures, non-pharmaceutical preparations, unpublished data, non-peer-reviewed reports, or outcomes with no plausible relationship to nutrition, metabolism, physical function, frailty, cachexia, or muscle-wasting disorders. In accordance with current best practice in ethnopharmacology, this review discusses pharmaceutical-grade Kampo formulations manufactured under standardized quality control consistent with the Japanese Pharmacopoeia and Japanese national regulation (23, 26). This standardization distinguishes prescribed Kampo formulations from heterogeneous herbal preparations and makes translational evaluation more tractable.

Where relevant, botanical and formulation reporting was aligned with the principles of the Consensus statement on the Phytochemical Characterisation of Medicinal Plant extracts (ConPhyMP), which emphasizes transparent reporting of starting plant materials, botanical drugs, source information, and extract or formulation characterization (27).

In addition, the Standards of Reporting Kampo Products (STORK) database was consulted as a formulation-transparency resource for regulated prescription Kampo products (28, 29). STORK provides product-characterization information, including Japanese Pharmacopoeia and package-insert information for Kampo extract products marketed in Japan. In this review, STORK was used to support transparent description and verification of Kampo formulations; it was not used as a substitute for peer-reviewed clinical evidence grading.

Because the review question spans heterogeneous evidence, findings were interpreted according to evidence proximity. Direct evidence refers to studies that explicitly assessed sarcopenia using established diagnostic criteria or measured core sarcopenia outcomes such as grip strength, physical performance, and muscle mass. Indirect evidence refers to studies that evaluated related domains such as appetite, frailty, fatigue, cachexia, inflammation, mitochondrial function, gut microbiota, neuropathy, or physical activity. This distinction is used throughout the review to avoid overinterpreting biological plausibility as clinically established efficacy.

## 3 Nutritional and metabolic drivers of sarcopenia

### 3.1 Chronic inflammation and catabolic signaling

Chronic low-grade inflammation, often termed inflammaging, plays a central role in the pathogenesis of sarcopenia. Aging is associated with increased circulating concentrations of inflammatory mediators such as interleukin-6, tumor necrosis factor-alpha, and C-reactive protein (13, 14). These mediators can activate intracellular pathways that favor muscle protein degradation through the ubiquitin-proteasome system, autophagy-lysosome pathways, and other catabolic processes (12–14, 30).

At the molecular level, inflammatory signaling may increase expression of muscle atrophy-related mediators such as atrogin-1 and muscle RING-finger protein-1, while also impairing satellite-cell function and muscle regeneration (12, 13, 30). These mechanisms are especially relevant in cachexia, but they may also contribute to muscle decline in frail older adults and chronically ill patients. Clinical studies have shown that higher levels of inflammatory markers are associated with longitudinal declines in muscle mass and strength, supporting the view that inflammation is not merely a correlate of aging but an active biological driver of muscle loss (13, 14).

### 3.2 Mitochondrial dysfunction and impaired energy metabolism

Mitochondrial dysfunction is another major contributor to skeletal muscle aging. Aging skeletal muscle exhibits reduced mitochondrial biogenesis, impaired oxidative phosphorylation, and increased production of reactive oxygen species (15–17). These alterations compromise cellular energy metabolism and promote oxidative damage to lipids, proteins, and mitochondrial DNA, ultimately contributing to muscle fiber atrophy and impaired contractile efficiency.

Because skeletal muscle is highly dependent on mitochondrial ATP production, even modest impairments in mitochondrial function may translate into clinically meaningful declines in endurance, strength, and physical performance. Mitochondrial dysfunction is therefore increasingly recognized as a central pathological node in sarcopenia biology and a compelling translational target (3, 15–17).

### 3.3 Anabolic resistance and impaired proteostasis

Anabolic resistance is defined as the reduced ability of aging skeletal muscle to stimulate protein synthesis in response to dietary amino acids or resistance exercise (17–19). In healthy younger adults, nutrient intake and mechanical loading activate anabolic pathways, including mechanistic target of rapamycin signaling. In contrast, older skeletal muscle exhibits a blunted anabolic response, making physiological stimuli less effective in preserving lean mass (17–19).

This shift toward reduced protein synthesis, combined with increased proteolysis, gradually drives net muscle catabolism. Reduced physical activity, chronic disease, insulin resistance, inflammation, and inadequate protein intake may further aggravate anabolic resistance in older adults (17–19). The clinical implication is that interventions aimed at appetite or oral intake may not be sufficient unless the biological context allows ingested nutrients to be translated into muscle maintenance.

### 3.4 Neuromuscular degeneration and functional decline

Loss of motor neurons and degeneration of neuromuscular junctions also contribute to age-related muscle decline (3). Denervation of muscle fibers leads to fiber atrophy and reduced force generation. Although compensatory reinnervation may occur through collateral sprouting of surviving motor neurons, this process becomes less effective with advancing age, accelerating muscle weakness and functional decline.

Importantly, sarcopenia is not defined by low muscle mass alone. Contemporary diagnostic frameworks, including the European Working Group on Sarcopenia in Older People 2 and the Asian Working Group for Sarcopenia, emphasize assessment of muscle strength, muscle quantity or quality, and physical performance (1, 4, 31–33). This perspective is essential when evaluating Kampo medicine, because potential benefits may occur through appetite, fatigue, activity tolerance, or neuromuscular symptoms rather than through a measurable direct increase in muscle mass.

## 4 Frailty, sarcopenia, and cachexia: distinctions and overlap

Although frailty, sarcopenia, and cachexia overlap, they should be distinguished conceptually and operationally. Sarcopenia is primarily a muscle disorder defined by reduced muscle strength and low muscle quantity or quality, with physical performance used to grade severity (1, 4). Frailty is a multidimensional vulnerability state reflecting reduced physiological reserve; the Fried frailty phenotype includes weakness, exhaustion, slow gait speed, low physical activity, and unintentional weight loss (10, 34). Cachexia is a disease-associated wasting syndrome driven by systemic inflammation, metabolic dysregulation, and reduced food intake, and is often incompletely reversible by nutritional support alone (7, 8, 11, 35).

The concept of a frailty-sarcopenia-cachexia continuum is clinically useful because older and chronically ill patients commonly move across these categories or fulfill criteria for more than one condition. However, the continuum should not be used to obscure diagnostic differences. A therapy that improves appetite or fatigue in frailty cannot automatically be considered a sarcopenia therapy unless it has been tested against validated sarcopenia endpoints. Conversely, an intervention that preserves physical activity or food intake may still be clinically meaningful if it supports exercise and nutritional therapy in multimodal care.

In this review, we therefore use the continuum as a translational framework rather than as proof of equivalence. Claims about Kampo medicine are framed according to whether the evidence concerns sarcopenia directly or adjacent domains such as frailty, cachexia, anorexia, fatigue, inflammation, metabolic dysfunction, or neuromuscular symptoms.

## 5 Kampo medicine as a standardized nutrition-relevant intervention

Kampo medicine consists of standardized herbal formulations composed of multiple crude drugs derived mainly from plants and, less commonly, from minerals and animal products (23). In modern Japanese clinical practice, Kampo medicines are manufactured under pharmaceutical quality control and prescribed within a contemporary biomedical setting. This regulatory standardization distinguishes pharmaceutical-grade Kampo from heterogeneous herbal preparations used in many other settings and is critical for reproducible clinical research. In this review, each formula is therefore treated as a regulated prescription Kampo formulation rather than as an undefined herbal mixture; its component crude drugs and official indications are listed explicitly to support reproducibility and interpretation by readers outside Japan.

The relevance of Kampo medicine to sarcopenia lies not only in its multi-component pharmacology but also in its potential to influence clinically important nutrition-related domains such as appetite, oral intake, fatigue, inflammatory burden, metabolic function, and activity tolerance (36–40). In older adults and chronically ill patients, these domains often determine whether nutritional support can be maintained and whether rehabilitation or exercise interventions can be implemented.

Recent developments in systems pharmacology and network pharmacology support the idea that multi-component medicines may influence multiple biological nodes simultaneously (26, 37–43). This concept is appealing in sarcopenia because inflammation, mitochondrial dysfunction, appetite dysregulation, anabolic resistance, physical inactivity, and neuromuscular decline interact at the systems level. However, systems pharmacology should not be used as a substitute for clinical evidence. It should be treated as a hypothesis-generating framework that requires validation using standardized endpoints and transparent reporting.

## 6 Representative Kampo formulas and evidence base

**Figure 2:**
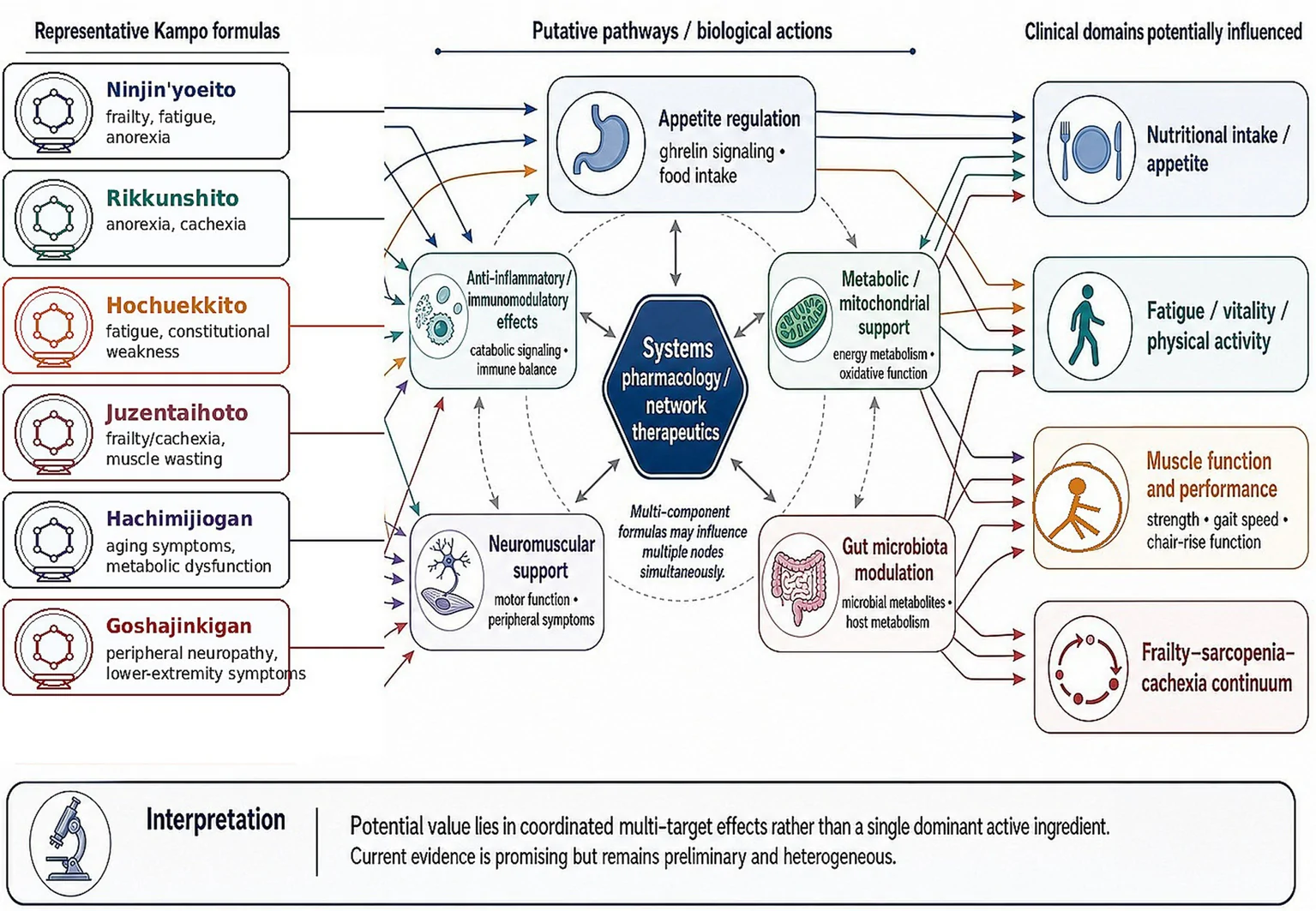
Nutrition-relevant actions of Kampo medicine in sarcopenia. Six selected representative Kampo formulas, including Juzentaihoto, may influence appetite regulation, oral intake, inflammatory and immunomodulatory pathways, mitochondrial and metabolic support, neuromuscular symptoms, and gut microbiota-related pathways. The figure uses non-botanical abstract icons for formulas and neutral functional-performance imagery rather than hyper-muscular icons. The figure should be interpreted as a hypothesis-generating framework rather than evidence that all formulas directly improve sarcopenia outcomes.

Representative Kampo formulas relevant to nutrition-related muscle-wasting disorders are summarized in Table 1, and the six selected representative formulas are illustrated in Figure 2. The table intentionally separates direct sarcopenia evidence from indirect evidence derived from frailty, cachexia, appetite, fatigue, inflammation, metabolism, or neuropathy studies. This distinction is necessary because the current literature is uneven across formulas and outcome domains. To support botanical and pharmacological transparency, the official crude-drug composition, representative botanical source names, traditional Kampo indication patterns, and authorized Japanese clinical indications of the six formulas are summarized in Table 2 on the basis of Japanese package-insert information, Japanese Pharmacopoeia nomenclature, and STORK-supported product characterization (28, 29, 44–48).

**Table 1:** Representative Kampo formulas and evidence proximity for nutrition-related relevance to muscle-wasting disorders.

Formula	Representative evidence	Population/exposure	Main findings relevant to nutrition or muscle	Interpretation for sarcopenia	Evidence level and key limitations	Evidence type/proximity
Ninjin’yoeito	Frailty and anorexia studies; open-label exploratory FRAMINGO study (*n* = 14; 24 weeks); preliminary open-label randomized controlled trial in COPD with frailty and sarcopenia (*n* = 53; 24 weeks) (49–53)	Older adults with frailty, anorexia, fatigue, cognitive impairment, or COPD with comorbid frailty/sarcopenia; exposure generally short-term to 24 weeks depending on study	Reported improvements in appetite, fatigue, frailty-related domains, quality of life, physical activity, and muscle-related outcomes in preliminary or open-label settings	Most directly relevant among the reviewed formulas, particularly for appetite, fatigue, activity, and multimodal support	Promising but still preliminary; several studies are open-label or disease-specific; larger blinded trials with prespecified EWGSOP2/AWGS outcomes are needed	Direct and indirect clinical evidence; preliminary sarcopenia-specific RCT evidence in COPD with frailty/sarcopenia.
Rikkunshito	Experimental and clinical cachexia/anorexia literature centered on ghrelin signaling (54–56)	Cancer-associated anorexia/cachexia and gastrointestinal appetite-related contexts; sarcopenia-specific populations rarely studied	Enhancement of ghrelin-related signaling and improvement of anorexia/food intake have been reported	Relevant to nutritional drivers of muscle wasting through appetite and oral intake rather than direct anabolic action	Indirect evidence for sarcopenia; requires trials linking appetite improvement to muscle strength, mass, and performance outcomes	Indirect clinical evidence for anorexia/cachexia plus preclinical/mechanistic evidence on ghrelin-related appetite pathways.
Hochuekkito	Clinical and experimental studies on immune modulation, systemic inflammation, nutritional status, fatigue, and COPD rehabilitation contexts (58–61)	Older or chronically ill patients with weakness, COPD, inflammation, or fatigue-related functional compromise	Reported immunomodulatory and anti-inflammatory effects; studies in COPD suggest possible relevance to inflammatory or nutritional status	Potentially relevant when inflammation, fatigue, low intake, and reduced activity tolerance contribute to muscle-wasting risk	Indirect evidence; limited sarcopenia-specific endpoints and heterogeneous study designs	Indirect clinical evidence in chronic disease/fatigue contexts plus preclinical or mechanistic immunomodulatory evidence.
Hachimijiogan	Experimental studies on metabolic regulation, insulin resistance, and oxidative stress (62, 63)	Mainly animal or metabolic models; aging-related symptoms and metabolic dysfunction in traditional clinical use	Potential effects on metabolic regulation, oxidative stress, and mitochondrial-related pathways	Mechanistically relevant to metabolic resilience and activity tolerance in aging muscle	Primarily biological plausibility; no robust sarcopenia-specific clinical trial evidence	Primarily preclinical/mechanistic evidence; sarcopenia-specific clinical evidence lacking.
Goshajinkigan	Retrospective clinical studies of oxaliplatin-induced peripheral neuropathy and an experimental rodent model (64–66)	Patients or models with peripheral neuropathy, lower-extremity symptoms, or oxaliplatin-induced neuropathy	Potential improvement or prevention of neuropathy-related symptoms; relevance to mobility and activity capacity	May indirectly support physical function and rehabilitation participation when neuropathic symptoms limit activity	Indirect evidence; outcomes focus on neuropathy rather than sarcopenia diagnostic criteria or muscle outcomes	Indirect clinical evidence for neuropathy-related function plus preclinical/mechanistic evidence; sarcopenia endpoints lacking.
Juzentaihoto	Preclinical SAMP8 sarcopenia-like aging model; KKAy obesity/type 2 diabetes model; prior diabetic muscle-atrophy and cachexia/anorexia-adjacent literature (67, 68)	Senescence-accelerated, diabetic, or metabolic mouse models; traditional use in constitutional weakness, fatigue, anorexia, anemia, and post-illness or postoperative decline	Suppression of muscle atrophy and motor-function decline; increased Sirt1 and IGF-1 expression; increased gastrocnemius wet weight and muscle-fiber cross-sectional area; reduced TNF-alpha, IL-6, atrogin-1, and MuRF1 in preclinical settings	Relevant as an additional Hozai candidate for inflammatory, oxidative, metabolic, and cachexia/anorexia-related muscle-wasting biology, but not established as a human sarcopenia therapy	Mainly preclinical/mechanistic and indirect evidence; human sarcopenia trials using standardized criteria and validated endpoints are lacking	Preclinical/mechanistic evidence plus indirect cachexia/anorexia-adjacent evidence; direct clinical sarcopenia evidence lacking.

**Table 2:** Official crude-drug composition, representative botanical sources, and clinical indications of the six Kampo formulas discussed in this review.

Formula	Constituent crude drugs: JP English names, amounts in source mixture, and representative botanical source(s)	Traditional Kampo indication/Sho	Official Japanese indication or modern clinical domain
Ninjin’yoeito	JP Rehmannia root 4.0 g (*Rehmannia glutinosa*); JP Japanese angelica root 4.0 g (*Angelica acutiloba*); JP Atractylodes rhizome 4.0 g (*Atractylodes japonica*/*Atractylodes macrocephala*); JP Poria Sclerotium 4.0 g (*Wolfiporia cocos*); JP Ginseng 3.0 g (*Panax ginseng*); JP Cinnamon bark 2.5 g (*Cinnamomum cassia*); JP Polygala root 2.0 g (*Polygala tenuifolia*); JP Peony root 2.0 g (*Paeonia lactiflora*); JP Citrus Unshiu Peel 2.0 g (*Citrus unshiu*); JP Astragalus root 1.5 g (*Astragalus mongholicus*/*Astragalus membranaceus*); JP Glycyrrhiza 1.0 g (*Glycyrrhiza uralensis*/*Glycyrrhiza glabra*); JP Schisandra fruit 1.0 g (*Schisandra chinensis*).	Qi and blood deficiency pattern; constitutional weakness after illness; fatigue, anorexia, cold extremities, night sweating; supportive formula for deficient, depleted patients.	Relief of declined constitution after recovery from disease, fatigue/malaise, anorexia, perspiration during sleep, cold limbs, and anemia.
Rikkunshito	JP Atractylodes lancea Rhizome 4.0 g (*Atractylodes lancea*/*Atractylodes chinensis*); JP Ginseng 4.0 g (*Panax ginseng*); JP Pinellia Tuber 4.0 g (*Pinellia ternata*); JP Poria Sclerotium 4.0 g (*Wolfiporia cocos*); JP Jujube 2.0 g (*Ziziphus jujuba*); JP Citrus unshiu Peel 2.0 g (*Citrus unshiu*); JP Glycyrrhiza 1.0 g (*Glycyrrhiza uralensis*/*Glycyrrhiza glabra*); JP Ginger 0.5 g (*Zingiber officinale*).	Spleen/stomach Qi deficiency; weak stomach, anorexia, epigastric fullness, easy fatigability, anemia or cold limbs in a deficient digestive pattern.	Relief of gastritis, gastric atony, gastroptosis, maldigestion, anorexia, gastric pain, and vomiting in patients with weak stomach, appetite loss and epigastric fullness.
Hochuekkito	JP Astragalus root 4.0 g (*Astragalus mongholicus*/*Astragalus membranaceus*); JP Atractylodes lancea Rhizome 4.0 g (*Atractylodes lancea*/*Atractylodes chinensis*); JP Ginseng 4.0 g (*Panax ginseng*); JP Japanese Angelica root 3.0 g (*Angelica acutiloba*); JP Bupleurum root 2.0 g (*Bupleurum falcatum*); JP Jujube 2.0 g (*Ziziphus jujuba*); JP Citrus unshiu peel 2.0 g (*Citrus unshiu*); JP Glycyrrhiza 1.5 g (*Glycyrrhiza uralensis*/*Glycyrrhiza glabra*); JP Cimicifuga rhizome 1.0 g (*Actaea simplex*/*Cimicifuga simplex*); JP Ginger 0.5 g (*Zingiber officinale*).	Qi deficiency and spleen hypofunction; central Qi deficiency with fatigue, poor digestive function, reduced vitality, spontaneous sweating, or prolapse-like symptoms.	Relief of symptoms/conditions in patients with delicate constitution, reduced digestive function and severe fatigability of limbs, including summer emaciation, reinforcement of physical strength after illness, anorexia, gastroptosis, common cold, hemorrhoid, prolapse-related conditions, impotence, hemiplegia and hyperhidrosis.
Hachimijiogan	JP Rehmannia root 6.0 g (*Rehmannia glutinosa*); JP Cornus fruit 3.0 g (*Cornus officinalis*); JP Dioscorea rhizome 3.0 g (*Dioscorea japonica*/*Dioscorea polystachya*); JP Alisma rhizome/tuber 3.0 g (*Alisma orientale*); JP Poria Sclerotium 3.0 g (*Wolfiporia cocos*); JP Moutan bark 2.5 g (*Paeonia* × *suffruticosa*); JP Cinnamon bark 1.0 g (*Cinnamomum cassia*); JP powdered processed Aconite root 0.5 g (processed *Aconitum carmichaelii*).	Kidney deficiency pattern in later life; lower back or lower-extremity weakness, coldness or alternating cold/hot sensations, dry mouth, urinary disturbance, and constitutional fatigue.	Relief of symptoms in patients with severe fatigue/malaise, decreased urinary output or increased urinary frequency, dry mouth, and alternating cold/hot feeling in the extremities, including nephritis, diabetes mellitus, impotence, sciatica, low back pain, beriberi, cystitis, prostatic hypertrophy and hypertension.
Goshajinkigan	JP Rehmannia root 5.0 g (*Rehmannia glutinosa*); JP Achyranthes root 3.0 g (*Achyranthes bidentata*); JP Cornus fruit 3.0 g (*Cornus officinalis*); JP Dioscorea rhizome 3.0 g (*Dioscorea japonica*/*Dioscorea polystachya*); JP Plantago Seed 3.0 g (*Plantago asiatica*/*Plantago depressa*); JP Alisma rhizome 3.0 g (*Alisma orientale*); JP Poria Sclerotium 3.0 g (*Wolfiporia cocos*); JP Moutan Bark 3.0 g (*Paeonia* × *suffruticosa*); JP Cinnamon bark 1.0 g (*Cinnamomum cassia*); JP powdered processed aconite root 1.0 g (processed *Aconitum carmichaelii*).	Kidney deficiency with water dysregulation and lower-extremity symptoms; easily fatigued, cold extremities, urinary disturbance, leg pain, numbness or low back pain.	Relief of leg pain, low back pain, numbness, blurred vision in older patients, pruritus, dysuria, frequent urination and edema in patients with decreased urine volume or polyuria, sometimes dry mouth, easy fatigue and cold extremities.
Juzentaihoto	JP Astragalus root 3.0 g (*Astragalus mongholicus*/*Astragalus membranaceus*); JP Atractylodes lancea rhizome 3.0 g (*Atractylodes lancea*/*Atractylodes chinensis*); JP Cinnamon bark 3.0 g (*Cinnamomum cassia*); JP Japanese Angelica root 3.0 g (*Angelica acutiloba*); JP Rehmannia Root 3.0 g (*Rehmannia glutinosa*); JP Ginseng 3.0 g (*Panax ginseng*); JP Peony root 3.0 g (*Paeonia lactiflora*); JP Poria Sclerotium 3.0 g (*Wolfiporia cocos*); JP Cnidium rhizome 3.0 g (*Cnidium officinale*); JP Glycyrrhiza 1.5 g (*Glycyrrhiza uralensis*/*Glycyrrhiza glabra*).	Qi and blood deficiency pattern; constitutional weakness after illness or surgery; fatigue, anorexia, anemia, night sweating, cold extremities, and depleted physical strength.	Relief of declined constitution after recovery from disease or after surgery, fatigue/malaise, anorexia, night sweating, cold limbs, and anemia.

JP, Japanese Pharmacopoeia. Amounts refer to the crude-drug source mixture reported in Japanese package-insert information for prescription Kampo formulations; extract content and daily granule dose differ by manufacturer and product. Botanical names are representative source plants corresponding to JP crude-drug nomenclature and are provided for interpretive transparency, not as independent authentication of each marketed lot. STORK was additionally used as a product-characterization resource for Japanese prescription Kampo formulations.

### 6.1 Ninjin’yoeito

Ninjin’yoeito is among the Kampo formulas most frequently discussed in relation to frailty, fatigue, anorexia, and age-related decline. It contains twelve herbal components, including ginseng, astragalus root, rehmannia root, and angelica root. Traditionally, it has been prescribed for patients with constitutional weakness, fatigue, poor appetite, and delayed recovery after illness.

Clinical studies suggest that Ninjin’yoeito may improve appetite, fatigue, physical activity, or frailty-related domains in older adults (49–52). For example, the open-label exploratory FRAMINGO study administered Ninjin’yoeito 6–9 g/day for 24 weeks to 14 prefrail or frail patients (9 with mild cognitive impairment and 5 with mild Alzheimer’s disease) and reported improvement in anorexia and frailty-related measures (51). More directly relevant to sarcopenia, a preliminary open-label randomized controlled trial in patients with chronic obstructive pulmonary disease and comorbid frailty and sarcopenia [53 patients with Global Initiative for Chronic Obstructive Lung Disease stage II or higher disease, randomized to 24-week continuous Ninjin’yoeito administration (*n* = 25) or a delayed-administration control group (*n* = 28)] reported improvements in quality of life, physical activity, skeletal muscle mass index, knee-extension strength, walking speed, and respiratory-related outcomes during Ninjin’yoeito administration (53). This trial is important because it moves beyond purely indirect frailty evidence, although its open-label design, disease-specific population, and preliminary nature limit generalizability.

From a nutrition-oriented perspective, Ninjin’yoeito is relevant because appetite, fatigue, and activity tolerance may influence whether older adults can maintain oral intake and participate in rehabilitation. Nevertheless, evidence remains insufficient to conclude that Ninjin’yoeito is an established treatment for sarcopenia. The most appropriate interpretation is that it is a candidate supportive intervention requiring larger controlled studies with prespecified sarcopenia endpoints.

### 6.2 Rikkunshito

Rikkunshito has been studied extensively in relation to appetite regulation and cancer-associated anorexia-cachexia. Experimental work has demonstrated that Rikkunshito can enhance ghrelin signaling through mechanisms involving ghrelin secretion and ghrelin receptor-related pathways (54–56). Because anorexia and reduced oral intake directly contribute to muscle wasting in chronic disease and aging, this pharmacological profile is highly relevant to nutrition-oriented sarcopenia care.

In cachexia settings, Rikkunshito has been reported to ameliorate anorexia and improve food intake (55–57). However, these data should not be overextended to sarcopenia. The most defensible claim is that Rikkunshito may target the appetite and oral intake component of muscle-wasting disorders, especially in patients with cachexia or anorexia. Whether these effects translate into preserved muscle mass, strength, or physical performance in sarcopenia remains to be determined.

### 6.3 Hochuekkito

Hochuekkito is traditionally used for fatigue, constitutional weakness, and reduced vitality. Experimental and clinical studies suggest that Hochuekkito may exert immunomodulatory or anti-inflammatory effects (58–60). Studies in chronic obstructive pulmonary disease have also discussed potential effects on systemic inflammation, nutritional status, or pulmonary rehabilitation-related outcomes (60, 61). These domains are relevant because inflammation, fatigue, and reduced activity frequently coexist with poor intake in frail and chronically ill patients.

The connection to sarcopenia, however, remains indirect. Hochuekkito has not been established as a sarcopenia therapy, and the available literature does not consistently use diagnostic criteria or core muscle outcomes. It should therefore be viewed as a candidate formula for patients in whom fatigue, inflammatory burden, low intake, and reduced activity tolerance contribute to muscle-wasting risk.

### 6.4 Hachimijiogan

Hachimijiogan is commonly prescribed for aging-related symptoms, lower-extremity weakness, cold sensitivity, and metabolic dysfunction. Experimental studies suggest that Hachimijiogan may influence metabolic regulation, oxidative stress, and mitochondrial-related pathways (62, 63). These properties are relevant because impaired energy metabolism may contribute to reduced activity tolerance and poor nutritional resilience in aging muscle.

Direct clinical evidence for Hachimijiogan in sarcopenia is currently lacking. Its relevance is supported primarily by biological plausibility and experimental evidence rather than robust sarcopenia-specific clinical trial data. Future research would need to define whether metabolic effects translate into measurable improvements in muscle strength, physical performance, body composition, or activity.

### 6.5 Goshajinkigan

Goshajinkigan is often used for peripheral neuropathy, lower-extremity symptoms, and metabolic disorders. Its relevance to sarcopenia lies primarily in its potential effects on neuromuscular symptoms and activity capacity rather than in direct anabolic action (64–66). Because functional decline can limit mobility, rehabilitation participation, and meal-related independence, interventions affecting this domain may have indirect nutritional and rehabilitative importance.

Retrospective clinical studies and an experimental rodent model of Goshajinkigan have mainly focused on oxaliplatin-induced peripheral neuropathy (64–66). These data are relevant to physical function but are not equivalent to sarcopenia evidence. Future studies should assess whether symptom relief can improve activity, gait speed, strength, or nutritional independence in populations with sarcopenia or frailty.

### 6.6 Juzentaihoto

Juzentaihoto is a core Hozai formulation traditionally used for constitutional weakness, fatigue, anorexia, anemia, and recovery after illness or surgery. It shares several crude drugs and conceptual indications with other tonifying formulas, but it has a distinct position in the literature on frailty-related weakness, inflammatory wasting, and cancer-associated anorexia/cachexia.

Preclinical evidence provides a biologically plausible link between Juzentaihoto and muscle-wasting biology. In senescence-accelerated SAMP8 mice, Juzentaihoto administration suppressed skeletal muscle atrophy and motor-function decline, with mechanistic findings suggesting increased Sirt1 and IGF-1 expression and reduced inflammatory/catabolic signaling (67). In KKAy mice with obesity and type 2 diabetes, Juzentaihoto increased gastrocnemius wet weight and muscle-fiber cross-sectional area, improved rotarod performance, decreased serum TNF-alpha and IL-6, improved insulin resistance-related indices, and reduced atrogin-1 and MuRF1 expression while increasing Sirt1 expression (68). These findings are relevant to inflammatory, oxidative, and metabolic drivers of muscle wasting.

Nevertheless, these studies should be interpreted as preclinical and mechanistic evidence rather than clinical proof of efficacy in human sarcopenia. Juzentaihoto should therefore be discussed as an additional Hozai candidate with relevance to the frailty-sarcopenia-cachexia continuum, particularly in phenotypes characterized by constitutional depletion, inflammation, metabolic dysregulation, anorexia, or cachexia, but not as an established sarcopenia-specific therapy.

## 7 Gut microbiota, nutrient handling, and energy sensing as a mechanistic bridge

**Figure 3:**
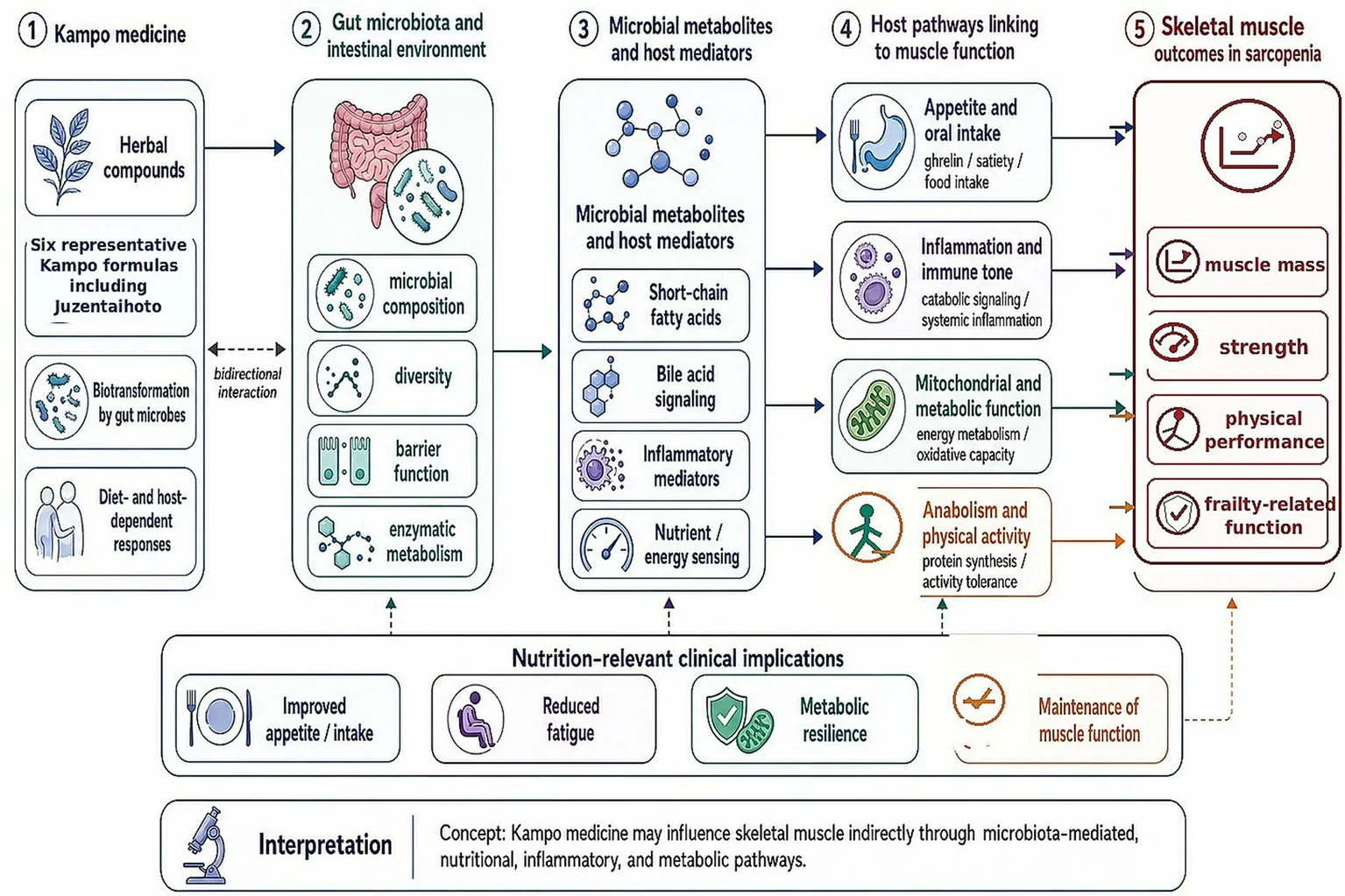
Gut–muscle axis linking Kampo medicine, nutrition-related pathways, and skeletal muscle function in sarcopenia. The Kampo medicine panel explicitly indicates six representative formulas, including Juzentaihoto. Herbal compounds and gut microbiota may interact bidirectionally, generating microbial metabolites and host mediators that influence appetite, inflammation, mitochondrial function, energy sensing, anabolism, physical activity, and muscle-related outcomes. Skeletal-muscle outcome icons were revised to avoid bicep-type imagery and to emphasize functional maintenance and measurement-oriented outcomes rather than supraphysiological enhancement. These pathways remain biologically plausible but require clinical validation using standardized sarcopenia endpoints.

The gut microbiota is increasingly recognized as a nutrition-relevant modulator of skeletal muscle health. Age-related alterations in microbial composition and diversity may influence nutrient handling, intestinal barrier integrity, inflammatory tone, insulin sensitivity, and host metabolic regulation, thereby affecting appetite, energy availability, and muscle-related function (69–74) (Figure 3). A 2025 systematic review and meta-analysis reported altered gut microbiota diversity and composition in patients with sarcopenia (10 studies; 421 patients with sarcopenia and 1,642 controls), including reduced alpha diversity together with a higher relative abundance of Proteobacteria and Escherichia-Shigella and a lower abundance of Firmicutes and Faecalibacterium, supporting the biological plausibility of a gut–muscle axis while also underscoring heterogeneity among studies (74).

Microbial metabolites such as short-chain fatty acids, bile acid-related signals, and other host-microbe mediators may influence nutrient sensing, mitochondrial metabolism, insulin sensitivity, and systemic inflammatory balance (69–74). These pathways provide a mechanistic link between intestinal ecology and clinically relevant sarcopenia domains, including oral intake, fatigue, activity tolerance, and skeletal muscle performance.

Herbal medicines interact extensively with the intestinal microbiota. Many phytochemicals are metabolized by gut bacteria into bioactive metabolites that influence host physiology, while herbal compounds themselves may reshape microbial composition and metabolic activity (69, 71–73, 75). In this context, Kampo medicine may influence skeletal muscle indirectly by modifying microbiota-related and nutrition-related pathways rather than by acting solely through direct anabolic effects on muscle tissue. This hypothesis is biologically plausible but remains insufficiently validated in sarcopenia-specific clinical trials.

## 8 Molecular anchoring of systems pharmacology

Systems pharmacology provides a conceptual framework for interpreting the biological effects of multi-component therapeutics such as Kampo medicine (26, 37–43). Rather than assuming a one-drug-one-target model, systems pharmacology conceptualizes disease as a network-level problem in which therapeutic agents modulate multiple nodes and pathways simultaneously. For sarcopenia, however, this framework must be anchored to specific biological pathways to avoid becoming a vague explanatory label.

Several pathway-level mechanisms are particularly relevant. First, inflammatory cytokines such as interleukin-6 and tumor necrosis factor-alpha may promote muscle catabolism through nuclear factor-kappa B activation, ubiquitin-proteasome signaling, and increased expression of atrophy-related mediators such as atrogin-1 and muscle RING-finger protein-1 (12–14, 30). A Kampo formula hypothesized to influence sarcopenia through anti-inflammatory effects should therefore be evaluated against inflammatory markers and catabolic signaling readouts, not only symptom scores.

Second, anabolic resistance involves impaired activation of nutrient- and exercise-responsive pathways, including mechanistic target of rapamycin and related protein synthesis pathways (17–19). An intervention that improves appetite or oral intake may have limited muscle benefit if anabolic responsiveness remains blunted. Trials should therefore combine dietary assessment with muscle strength, physical performance, and body composition outcomes.

Third, appetite-related mechanisms are particularly relevant to Rikkunshito because the formula has been linked to ghrelin signaling (54–56). This makes it mechanistically plausible for anorexia and cachexia, but sarcopenia trials would need to demonstrate that appetite effects translate into improved intake and muscle-related outcomes.

Fourth, microbiota-mediated metabolism may connect Kampo compounds to host energy sensing, bile acid signaling, short-chain fatty acid production, inflammatory tone, and mitochondrial function (69–74). This provides a coherent gut–muscle framework, but the direction and clinical magnitude of these effects are likely context-dependent. Accordingly, microbiome and metabolomic studies should be integrated with validated sarcopenia endpoints rather than interpreted as stand-alone evidence of clinical efficacy.

## 9 Clinical implications and evidence grading in multimodal sarcopenia management

From a clinical perspective, the potential value of Kampo medicine in sarcopenia may lie less in direct reversal of muscle loss and more in its ability to influence nutrition-relevant domains that contribute to muscle decline. These domains include appetite, oral intake, fatigue, physical activity tolerance, metabolic resilience, inflammatory balance, and neuromuscular symptoms. Such effects may be meaningful in older adults with multimorbidity, in whom sarcopenia frequently coexists with frailty, anorexia, fatigue, and reduced daily activity (76).

Within multimodal sarcopenia care, Kampo medicine may be positioned as a complementary supportive intervention alongside exercise, protein and energy optimization, rehabilitation, and management of underlying disease. This positioning is important because the clinical burden of sarcopenia is often driven not only by impaired muscle anabolism but also by reduced intake, poor tolerance of activity, and systemic physiological vulnerability.

At the same time, current evidence is insufficient to support broad recommendations for Kampo medicine as a standard sarcopenia therapy. Most available studies are small, open-label, observational, disease-specific, experimental, or focused on symptom domains such as anorexia and fatigue rather than on validated sarcopenia endpoints. Therefore, the evidence should be described as promising but preliminary, and conclusions should remain conditional on future validation.

The addition of Juzentaihoto further reinforces the need to distinguish evidence proximity across formulas. Ninjin’yoeito currently has the most direct clinical relevance among the reviewed formulations because preliminary studies have included frailty and sarcopenia-related outcomes, whereas Juzentaihoto is supported mainly by preclinical and cachexia/anorexia-adjacent evidence (67, 68). Recent formula-specific reviews in Traditional & Kampo Medicine provide useful background for individual prescriptions, but they do not eliminate the need for sarcopenia-specific trials using standardized diagnostic and functional endpoints (24, 25).

## 10 Safety, adverse events, and herb-drug interactions

Any discussion of Kampo medicine in older adults must include safety. Although Kampo formulations are widely prescribed in Japan, they are not risk-free. Older adults with sarcopenia or frailty often have multimorbidity, polypharmacy, renal impairment, low albumin, constipation, and reduced physiological reserve, all of which may increase vulnerability to adverse events.

A major safety concern is glycyrrhiza-related pseudoaldosteronism. Licorice-containing Kampo formulas can cause hypertension, edema, hypokalemia, myopathy, rhabdomyolysis, and arrhythmia through inhibition of 11β-hydroxysteroid dehydrogenase type 2 by glycyrrhizin metabolites (77, 78). Reported risk factors include higher daily glycyrrhiza exposure, long-term use, constipation, hypoalbuminemia, older age, hyperbilirubinemia-related transporter dysfunction, and concomitant medications such as diuretics (77, 78). These risks are directly relevant to sarcopenia because hypokalemia and myopathy may worsen weakness and functional decline.

Other clinically important adverse events include liver dysfunction, interstitial pneumonia, allergic reactions, gastrointestinal symptoms, and formula-specific effects related to crude drug composition. Potential herb-drug interactions should be considered, particularly in patients receiving diuretics, antihypertensives, anticoagulants, antiplatelet agents, corticosteroids, anticancer therapies, or multiple Kampo formulas from different prescribers.

Clinical implementation should therefore include careful formula selection, avoidance of unnecessary duplication, medication reconciliation, monitoring of serum potassium and liver enzymes when appropriate, attention to symptoms such as edema or weakness, and discontinuation or dose adjustment when adverse events are suspected. Safety monitoring should be built into future trials rather than treated as an afterthought.

## 11 Limitations of the current evidence base

Several limitations must be emphasized. First, the number of sarcopenia-specific clinical trials of Kampo medicine remains small. Many studies evaluate frailty, appetite, cachexia, chronic disease symptoms, neuropathy, or fatigue rather than sarcopenia defined by established criteria. Second, study designs are heterogeneous, and many reports are observational, open-label, or exploratory. Third, outcome measures vary widely across studies and frequently do not include core sarcopenia endpoints such as grip strength, gait speed, chair-stand performance, appendicular lean mass, or imaging-based muscle quality.

Fourth, mechanistic evidence is often derived from experimental models or adjacent disease states, making direct clinical translation uncertain. Fifth, formula composition, dose, duration, adherence, concomitant nutritional therapy, exercise exposure, and background disease severity are not always reported in sufficient detail. Finally, publication bias and selective emphasis on positive findings are possible in the Kampo literature, making balanced interpretation essential.

For these reasons, Kampo medicine should currently be presented as a hypothesis-generating and potentially supportive approach, not as an established disease-modifying therapy for sarcopenia. The central scientific task is to determine which formula, in which phenotype, at what stage of frailty or muscle wasting, and in combination with which nutritional or rehabilitation strategy, can produce clinically meaningful benefit.

## 12 Translational priorities and future directions

**Figure 4:**
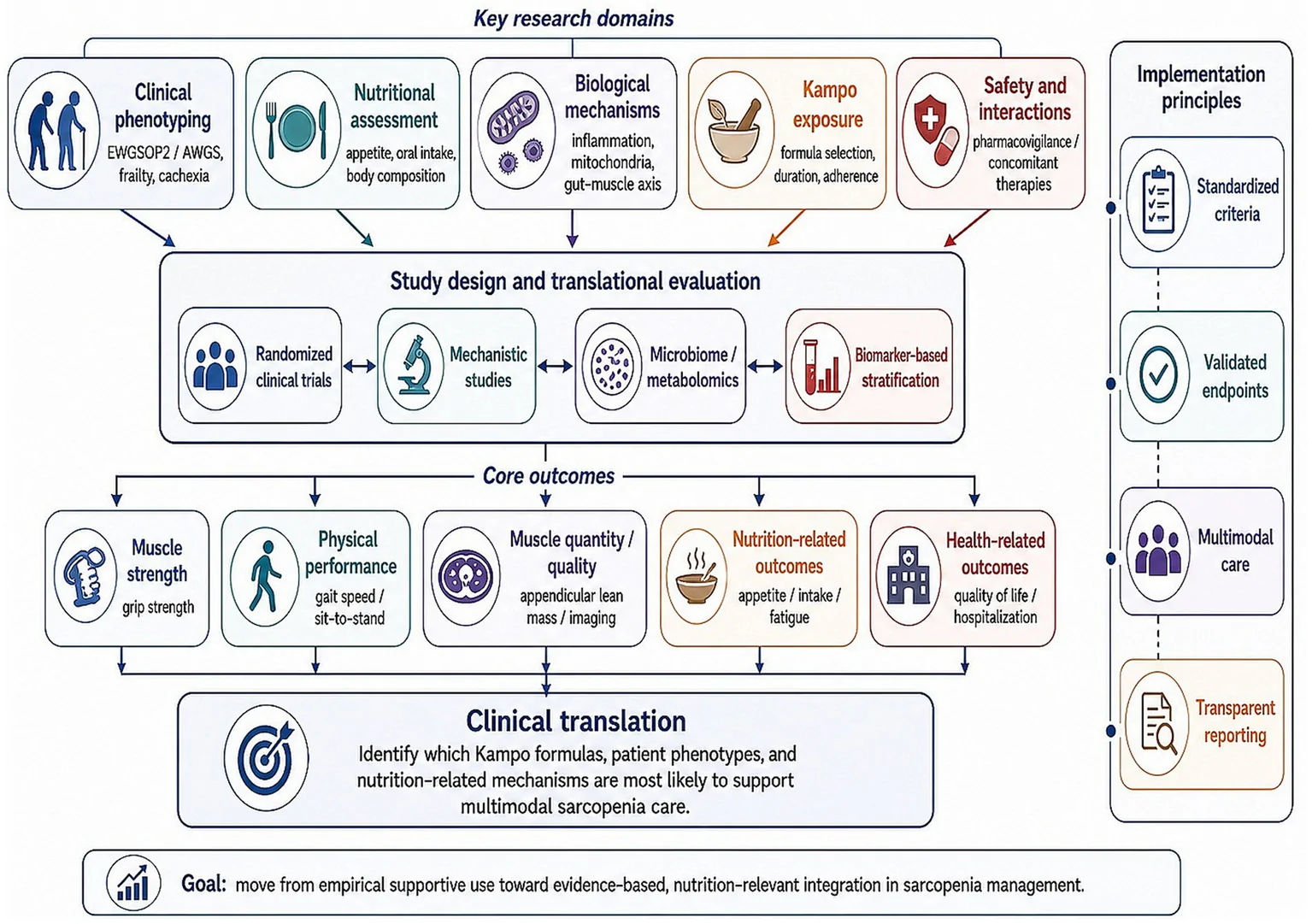
Translational research framework for evaluating Kampo medicine as a nutrition-relevant intervention in sarcopenia. Future studies should integrate standardized clinical phenotyping, nutritional assessment, biological mechanisms, Kampo exposure characterization, safety monitoring, randomized clinical trials, mechanistic studies, microbiome and metabolomic analyses, biomarker-based stratification, validated endpoints, and transparent reporting.

Future research should move beyond descriptive discussions of Kampo formulations and focus on mechanism-oriented and clinically translatable approaches (Figure 4). First, well-designed studies are needed to evaluate whether Kampo-based interventions can improve appetite, oral intake, adherence to nutritional support, fatigue, activity, and functional outcomes in patients with sarcopenia or related muscle-wasting states.

Second, trials should use standardized sarcopenia criteria such as the European Working Group on Sarcopenia in Older People 2 or the Asian Working Group for Sarcopenia together with validated outcomes including grip strength, five-times sit-to-stand performance, gait speed, appendicular lean mass, dietary intake, fatigue, and health-related quality of life (1, 4, 5, 22, 31). This is particularly important when interventions are intended to support multimodal care rather than act as stand-alone anabolic therapies.

Third, integration of microbiome research, metabolomics, mitochondrial phenotyping, inflammatory profiling, and nutritional phenotyping may provide deeper insights into mechanisms linking Kampo medicine, nutrient handling, energy sensing, and skeletal muscle metabolism (26, 37–41, 43, 69–74). Such interdisciplinary approaches may identify biomarkers of response and help define which patient populations are most likely to benefit from specific formulations.

Fourth, safety and implementation outcomes should be evaluated prospectively. Future studies should report formula composition, dose, duration, adherence, adverse events, serum potassium, liver function, concomitant medications, exercise exposure, nutritional intake, and withdrawal reasons. Transparent reporting will be essential if Kampo medicine is to move from empirical supportive use toward evidence-based integration into sarcopenia care.

## 13 Conclusion

Sarcopenia and related muscle-wasting conditions represent major challenges in aging societies. Because skeletal muscle decline is shaped not only by impaired anabolism but also by anorexia, reduced intake, metabolic dysregulation, mitochondrial dysfunction, inflammation, neuromuscular impairment, and inactivity, effective care often requires more than a single-pathway strategy.

Kampo medicine, with its standardized multi-component formulations, may provide a nutrition-relevant complementary approach capable of influencing appetite, oral intake, fatigue, inflammatory balance, metabolic regulation, neuromuscular symptoms, and gut–muscle axis pathways. The reviewed formulas, including Juzentaihoto as an additional Hozai candidate, should be interpreted according to evidence proximity: some have preliminary clinical relevance to frailty- or sarcopenia-related outcomes, whereas others are supported mainly by preclinical, mechanistic, or adjacent cachexia/anorexia evidence. These domains are clinically relevant because they help determine whether older adults can maintain nutritional intake and participate in multimodal interventions aimed at preserving muscle function.

At present, however, Kampo medicine should not be described as an established therapy for sarcopenia. Its potential relevance lies in hypothesis-generating, coordinated modulation of nutrition-related and function-related domains within multimodal care. Rigorous clinical studies using standardized sarcopenia criteria, validated endpoints, transparent reporting, and systematic safety monitoring are required to define its role in sarcopenia and related muscle-wasting disorders.
